# Designing Deferoxamine-Loaded Flaxseed Gum and Carrageenan-Based Controlled Release Biocomposite Hydrogel Films for Wound Healing

**DOI:** 10.3390/gels8100652

**Published:** 2022-10-14

**Authors:** Tayyaba Sadiq, Syed Haroon Khalid, Ikram Ullah Khan, Hira Mahmood, Sajid Asghar

**Affiliations:** Department of Pharmaceutics, Faculty of Pharmaceutical Sciences, Government College University Faisalabad, Faisalabad 38000, Pakistan

**Keywords:** biocomposites, wound healing, hydrogel film, deferoxamine, biocompatible

## Abstract

In this study, biocomposite hydrogel films made from flaxseed gum (FSG)/kappa carrageenan (CGN) were fabricated, using potassium chloride as a crosslinker and glycerol as a plasticizer. The composite films were loaded with deferoxamine (DFX), an iron chelator that promotes neovascularization and angiogenesis for the healing of wounds. The properties of the biocomposite hydrogel films, including swelling, solubility, water vapor transmission rate, tensile strength, elongation at break, and Young’s modulus studies, were tested. The films were characterized by scanning electron microscopy (SEM), Fourier transform infrared spectroscopy (FTIR), thermogravimetric analysis (TGA), and differential scanning calorimetry (DSC). In addition, drug release studies in PBS at pH 7.2 were investigated. In vivo analysis was performed by assessing the wound contraction in a full-thickness excisional wound rat model. Hematoxylin & eosin (H & E) and Masson’s trichome staining were performed to evaluate the effect of the films on wound healing progress. The visual and micro-morphological analysis revealed the homogenous structure of the films; however, the elongation at break property decreased within the crosslinked film but increased for the drug-loaded film. The FTIR analysis confirmed the crosslinking due to potassium chloride. A superior resistance towards thermal degradation was confirmed by TGA for the crosslinked and drug-loaded films. Drug release from the optimum film was sustained for up to 24 h. In vivo testing demonstrated 100% wound contraction for the drug-loaded film group compared to 72% for the pure drug solution group. In light of the obtained results, the higher potential of the optimized biocomposite hydrogel film for wound healing applications was corroborated.

## 1. Introduction

Wound healing is a dynamic and complex process that consists of four successive and overlapping cellular and biochemical processes: homeostasis, inflammation, proliferation, and remodeling. These processes tend to restore the anatomical structure and functionality of the skin [1]. These cascades of cellular and biochemical events occur between the extracellular matrix (ECM) and the different cellular constituents of the skin. If the process of wound healing is interrupted due to any physiological or pathological condition, it may lead to the development of ulcers and the excessive formation of scars or keloids. Unfortunately, there is currently no effective treatment to counteract these pathological challenges. One major hurdle is poor understanding of the cellular and molecular mechanisms of wound healing, and thus this affects the development of effective new treatments for wounds [2].

Wound healing is not only a tenacious issue for clinicians, but it also puts a heavy burden on patients, both financially and physically. Conventional wound care models include skin grafting, laser therapy, and skin flap transplantation; however, all of these present risks of atrophic scars and pigmentary abnormalities [3]. Recent research into polymers has seen considerable efforts devoted to the development of hydrogels intended for wound healing [4].

Deferoxamine (DFX), an FDA-approved iron chelator, is used clinically for the removal of excess iron in conditions such as beta thalassemia and intracerebral hemorrhage. It is also used as a hypoxia-mimicking agent [5]. Due to the exceptional properties of DFX, including chelation, it has been widely studied in the past decade. [6]. Some studies have reported the use of DFX to induce angiogenesis in bone and skin regeneration. These studies publicized that DFX induces the hypoxia-inducible factor-1 alpha (HIF-1α) and then stimulates the expression of the vascular endothelial growth factor (VEGF) and stromal cell-derived factor-1alpha (SDF-1α) to accelerate blood vessel formation [7]. DFX-treated wounds showed improved neogenesis, a reduction in free radical formation, an increase in collagen density, and decreased cell death. These findings suggest that DFX not only improves wound healing when applied topically but also reduces scar formation [8]. Despite its usefulness, DFX has a shorter half-life and associated systemic toxicity; studies have reported that elderly patients are particularly at an increased risk for a number of serious systemic reactions when DFX is consumed orally [9]. Hence, there is a need for a suitable vehicle for the controlled local delivery of DFX.

Natural polymers are biocompatible, non-toxic, versatile, and can be modified according to the requirements of the dosage due to the presence of different functional groups in their structure. Natural polymers are extensively investigated for their use in regenerative medicines due to its structural similarities with the body, such as ECM and connective tissues. A large number of studies have been carried out utilizing natural polymers in regenerative medicine and investigating the relationship between polymer properties and their intended physical forms, such as hydrogels, fibers, foams, microspheres, and sponges. Composites formed from biopolymers, such as proteins and polysaccharides, are considered substitutes for non-biodegradable polymers for different applications, such as medicinal use, food packaging, and even plastic bag production due to their low impact on the environment [10].

Flaxseed (*Linum usitatissimum* L.) gum (FSG), a heterogeneous polysaccharide, is composed of galacturonic acid (21.0–36.0%), xylose (19.0–38.0%), galactose (12.0–16.0%), rhamnose (11.0–16.0%), arabinose (8.0–13.0%), and glucose (4.0–6.0%). It also contains acidic and neutral fractions; the acidic fraction contains two sub-fractions of a molecular mass of 650 and 17 kDa, while the neutral fraction contains arabinoxylans with b-D-(1, 4)-xylan chains with a molecular mass of 1200 kDa [11]. FSG hydrogel films do not possess sufficient mechanical strength, so other polymers are usually blended to obtain the desired properties. PVA-FSG films showed improvement in mechanical strength via mutual blending [12]. FSG physicochemical properties were also reported to be improved by the addition of agar and cellulose nanocrystals. However, the cytotoxic glutaraldehyde was employed as the crosslinker [13].

Carrageenan (CGN) is an ideal material for wound healing, drug delivery, and tissue engineering applications [14]. It is a linear, sulfated, and hydrophilic polysaccharide that consists of repeating units of (3,6)-anhydro galactose and galactose. These repeating units are connected by alternating α-(1,3)- and β-(1,4)-glycosidic links [15]. Algal polysaccharides are widely being used in drug delivery systems due to a multitude of beneficial biological effects, e.g., (i) antibacterial, antiviral, and antifungal activity, (ii) a reduction in oxidative stress and inflammation, (iii) helpful in regulation of hemostasis and coagulation, (iv) and the modulation of angiogenic activity and immune response [16]. There are different types of CGN; out of these types, kappa-CGN undergoes gelation in the presence of potassium ions, and it forms strong and rigid gels, while other CGN types produce elastic, dry, and soft gels [17]. Due to the thixotropic behavior of CGN, it is considered most suitable for inclusion in other macromolecules [18].

In this study, biopolymers, FSG and CGN, were used in combination to design biodegradable biocomposite hydrogel films with improved mechanical, physical, chemical, and biological properties. These films were loaded with DFX for its controlled delivery at the wound site to exploit its wound healing potential. The hydrogel films were subjected to swelling, solubility, water vapor transmission rate, tensile strength, and elongation at break studies to understand their physicochemical and mechanical behavior. The films were also characterized by scanning electron microscopy (SEM), Fourier transform infrared spectroscopy (FTIR), thermogravimetric analysis (TGA), and differential scanning calorimetry (DSC). The ability of the hydrogel films to control the drug release was investigated in PBS at pH 7.2. In vivo analysis was performed by assessing the wound contraction in a full thickness excisional wound rat model.

## 2. Results and Discussion

### 2.1. Preparation of Biocomposite Hydrogel Films

**Figure 1:**
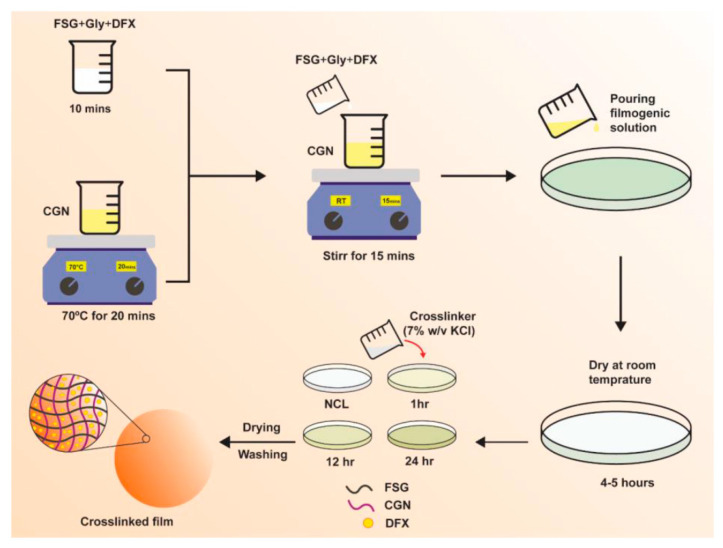
Schematics of DFX-loaded FSG/CGN biocomposite hydrogel film.

Biocomposite hydrogel films of FSG and CGN were prepared and crosslinked with a potassium chloride (KCl) solution via a casting method. The scheme of study is shown in Figure 1, and the composition of the preparations is given in Table 1. Initially, the different blends of FSG and CGN were prepared, including F_0.5_C_0.75_, F_1_C_0.5_, F_1_C_1_, F_1.5_C_0.5_, F_2_C_2_, C_0.5_F_0.75_, C_1_F_0.5_, and C_1.5_ F_0.5_; however, only F_1_C_1_, F_1.5_C_0.5_, and C_1.5_ F_0.5_ were selected due to homogenous film-formation, ease of peeling, absence of any fractures and deformities, and the integrity of the films in the PBS medium for more than an hour.

All composites were transparent and flexible. However, after crosslinking, there was a slight change in color from transparent to white. This change in color was also reported for a CGN-based functional hydrogel film that was reinforced with sulfur nanoparticles and grapefruit seed extract [19]. A subjective evaluation was carried out for the composites through visual and tactile observations. Only films that were homogenous (uniformity in color, absence of any insoluble material), continuous (absence of any rupture or brittle zones), and smooth (easy to handle and use) were selected. Composites lacking these characteristics were disposed off. This visual evaluation was carried out as reported elsewhere [20,21]. The films of CGN and FSG were crosslinked by varying the ratios of KCl (1, 3, 5, 7, and 10% *w*/*v*) with different immersion times. The films made with less than 7% *w*/*v* KCl solution had poor integrity and dissolved within less than an hour in the medium, whereas the films crosslinked with more than 7% *w*/*v* KCl were rigid, coarse, and lacked a homogenous appearance due to white residual precipitates. Therefore, the 7% *w*/*v* KCl solution was selected for the preparation of the biocomposite films. The crosslinking of CGN with KCl has been reported to promote a coli-to-helix formation, leading to a more rigid and firmer structure [22].

### 2.2. Film Thickness and Weight Variation

The thickness of the hydrogel films is shown in Table 2. While designing a wound dressing, a thickness less than that of human skin is desirable and preferred. The thickness of human dermis varies from 0.5 mm to 2.0 mm and depends upon different physiological and pathological factors [23]. Upon exposure to an aqueous medium, the hydrogels swell and increase in thickness and weight by imbibing the water; thus, the lesser thickness of the hydrogel films (as a dressing at the wound bed) would be beneficial to allow the maximum absorption of the wound exudates by compensating for the thickness. All the non-crosslinked composites had a similar thickness irrespective of the ratio of the polymer. The crosslinking affected film thickness and the greatest thickness was observed for the C_1.5_F_0.5_ biocomposites (0.17–0.18 mm), owing to the maximum involvement of CGN in the crosslinking. An increase in thickness due to crosslinking was also observed for the alginate films crosslinked with calcium ions [24].

The lowest thickness, after crosslinking (0.07 mm), was observed for F_1.5_C_0.5_ due to a lower ratio of CGN (0.5%). The prolonged exposure of these composites to the crosslinker (of up to 24 h) resulted in an increase in thickness to 0.12 mm due to the crosslinking of all available CGN. The F_1_C_1_ biocomposites showed an intermediate range of thickness, i.e., 0.11, 0.13, and 0.17 mm for the 1, 12, and 24 h crosslinked films, respectively. These findings are in line with the already reported chitosan-montmorillonite [25] and gelatin films [26].

The uniformity of the films was determined in terms of weight content. All films were found to be uniform in weight despite the varying polymers ratios (Table 2) and showed no significant variation (*p* > 0.05). Slight weight variations in the crosslinked films (as opposed to the non-crosslinked films) were due to the dissolution (crosslinker solution) of surface polymeric chains; however, these changes were not statistically significant, as communicated by Shahzad et al., 2018 [27].

### 2.3. Water Solubility (WS)

The neat biocomposite films without a crosslinker showed 100% solubility (Table 2), suggesting no crosslinking in the polysaccharide chains; however, the crosslinked films showed a significant reduction in solubility. These results are in line with previous reports [13]. The reduction in WS in the crosslinked film is indicative of the stability and resistance of the formulation to water. The addition of a crosslinker decreased solubility up to 60% for F_1_C_1_. This reduction in solubility was due to KCl, which causes rigidity by crosslinking the polysaccharide chains. F_1.5_C_0.5_ showed a solubility reduction of up to 75% after 12 h of immersion. This reduction is comparatively less due to the high concentration of FSG, imparting higher hydrophilicity to chains; however, 24 h of immersion in KCl might have crosslinked all of the chains of the polysaccharides, thus dropping the solubility to 59%. Comparable values for solubility were previously reported by Agudelo et al., 2020 [20]. The least soluble film was the C_1.5_F_0.5_ films due to a high content of CGN, where solubility was reduced to 49%, indicating a tighter 3D network formation in these hydrogel films.

### 2.4. Water Vapor Transmission Rate (WVTR)

WVTR is an important parameter to be analyzed along with solubility for hydrogel films that are intended for wound healing applications. WVTR is an indicator of the permeation of gasses and moisture through the films. The values of WVTR must be optimal, neither too high nor too low. The WVTR values for F_1_C_1_ were 859, 764, 732, and 1188 g/m^2^.day for the control, 1, 12, and 24 h crosslinking, respectively. These findings for WVTR are in line with those reported for gelatin/chitosan films [28]. F_1.5_C_0.5_ showed a higher WVTR than F_1_C_1_ (Table 2) due to a high ratio of the highly hydrophilic FSG, as reported by Giz and coworkers for alginate films [29]. The C_1.5_F_0.5_ composites showed comparatively lower WVTR values due to a higher ratio of CGN. These values correspond well with those reported for crosslinked films using sodium alginate and pectin [27]. A wide range of WVTR values have been reported for effective wound healing applications, and these values lie in the acceptable range. WVTR decreases due to the crosslinking process. Moreover, increasing the immersion time in the crosslinker or increasing the crosslinker concentration also decreases the WVTR [30]. However, F_1_C_1_ showed the highest WVTR after 24 h of immersion. This could be attributed to the 3D crosslinked mesh made by CGN in such a way that it allowed the FSG to expand within the dimensions of the film without losing its integrity.

### 2.5. Swelling Studies

**Figure 2:**
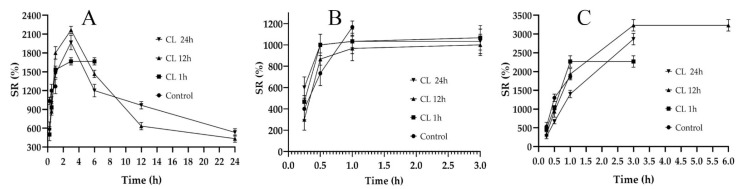
Swelling studies of the non-crosslinked (Control) and crosslinked (CL) films for 1, 12, and 24 h immersion. (**A**) F_1_C_1_, (**B**) F_1.5_C_0.5,_ and (**C**) C_1.5_F_0.5_. Data presented as mean ± SD.

A hydrogel film’s ability to absorb biological fluid is an important parameter, as it is responsible for the dissolution and release of the active ingredients entrapped in the polymeric network [31]. Swelling studies are often carried out to compare water absorption capacity, which is similar to the absorbing wound exudate [32]. Swelling ratio (SR) is also indicative of the degree of crosslinking in the composites [33]; therefore, it is important to design the films while controlling the crosslinking. The SRs of the fabricated films are shown in Figure 2. In Figure 2A, the non-crosslinked formulation (control) of F_1_C_1_ showed a maximum swelling up to 1 h of immersion and degraded afterwards. The lack of crosslinking and the presence of hydrophilic FSG is responsible for this abrupt swelling and degradation, as observed by Bergonzi et al. with their 2020 alginate/elastin film [34]. An analogous abrupt swelling behavior was also reported for cellulose/FSG composite hydrogels [35]. The crosslinked (CL) films of F_1_C_1_ showed a maximum swelling up to 4 h after immersion and remained intact for 24 h without visible signs of disintegration. This decrease in SR could be due to its high crosslinking density, as the same phenomenon was reported for citric acid crosslinked CMC-HPMC hydrogel films [34]. Figure 2B depicts the lowest swelling for F_1.5_C_0.5,_ which lasted for 3 h. In Figure 2C, C_1.5_F_0.5_ crosslinked for 12 h and displayed the highest SR of all the biocomposite films but only lasted for 6 h and disintegrated afterward due to the insufficient crosslinking of the chains. Hence, F_1_C_1_.12 was selected as the optimum composite film with appropriate ratios of polymers and a crosslinking density that permits reasonable swelling, integrity, WS, and WVTR.

### 2.6. Encapsulation Efficiency (EE%)

The results of the EE of DFX in the biocomposite hydrogel film (F_1_C_1_.12) are presented in Table 3. DFX (1%) transdermal patches have been reported for diabetic ulcer healing [9]. In our study, we observed that 1% loading yielded the maximum EE, whereas lower or higher loadings of the drug resulted in a loss of the drug. Therefore, we selected the 1% loaded DFX film (F_1_C_1_.DFX) for further studies.

### 2.7. Mechanical Properties of Hydrogel Films

The folding endurance (FE), tensile strength (TS), elongation at break (EAB), and Young’s modulus (YM) were determined for all films, as shown in Table 4. Wound dressings should possess good TS, high EAB, and low YM for their application and handling purposes to indicate durability and stress resistance. The blank and drug-loaded films displayed good folding endurance. The TS was higher for F_1_C_1_.12 and F_1_C_1_.DFX than for F_1_C_1_ due to crosslinking, as reported in the literature [36]. The EAB decreased for F_1_C_1_.12 when compared to F_1_C_1_, which indicates that the flexibility was lower upon crosslinking. Interestingly, F_1_C_1_.DFX showed a massive improvement in its EAB, which has been attributed in the literature to an increase in the plasticity of the films upon the loading of the payloads [26]. The lower YM for the crosslinked and drug-loaded films shows the increased rigidity and stiffness imparted by a higher degree of crosslinking [37]. Although the values of YM for all the composites were lower, these are still comparable to commercial dressings, such as the YM of the Kaltostat^®^ (ConvaTec, Reading, UK), Aquacel^®^ Ag (ConvaTec, Reading, UK), Aquacel^®^ Extra (ConvaTec, Reading, UK), and Exufiber^®^ (Mölnlycke, Göteborg, Sweden), which vary from 0.24 to 0.95 MPa. Mepilex^®^ (Mölnlycke, Göteborg, Sweden) and Mepilex^®^ Ag (Mölnlycke, Göteborg, Sweden) have the lowest YM but the highest total elongation, which signifies their flexibility [38]. In this study, F_1_C_1_.DFX displayed a lower YM, the highest EAB, an appropriate TS, and sufficient FE; hence, we can infer that F_1_C_1_.DFX possesses sufficient mechanical strength for application as a wound dressing [38].

### 2.8. Scanning Electron Microscopy

**Figure 3:**
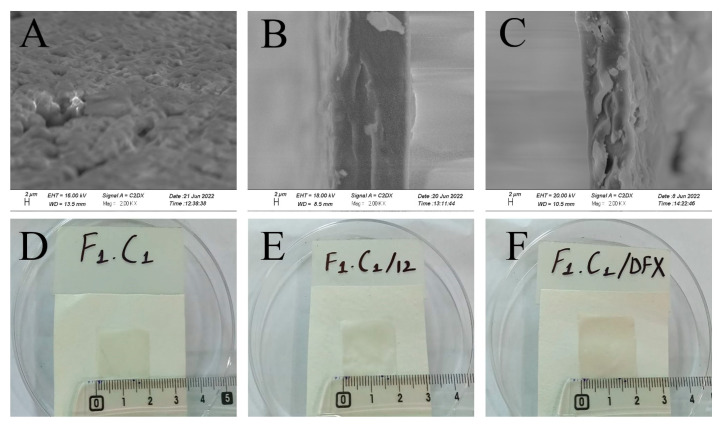
Cross-sectional SEM images of (**A**) F_1_C_1_, (**B**) (F_1_C_1_.12), (**C**) (F_1_C_1_.DFX), and photographs of the respective composites (**D**–**F**), respectively.

SEM analysis was used to study and understand the morphology of the control, crosslinked, and drug-loaded hydrogel films. It is evident from Figure 3A that F_1_C_1_ showed semi-interpenetrating hydrogel composite morphology. This reveals the proper mixing of FSG and CGN in the hydrogel film, showing good compatibility as matrix-forming polymers. This cross-sectional view also reveals a swollen 3D porous structure due to the hydrophilic groups in the film. In an earlier study, an interpenetrating hydrogel was reported for polyvinyl alcohol/chitosan/AgNO_3_/vitamin crosslinked composites [36]. Figure 3B,C display more compact structures as KCl facilitates intermolecular interaction and crosslinking. The increased roughness and sightly crystallized formation of the film could be attributed to a decrease in solubility; this has been reported for composites of alginate and babassu coconut mesocarp [21]. White protuberances through the cross section of the composite in Figure 3C could be referred to as the embedding of DFX within the hydrogel matrix, which is in good agreement with the findings of Li et al., 2020, for gelatin films. [26]. The visual appearances were the same for the non-crosslinked, crosslinked blank, and drug-loaded films, as shown in Figure 3D–F, respectively. All films were transparent without any signs of obvious differences.

### 2.9. Fourier Transform Infrared Spectroscopy (FT-IR)

**Figure 4:**
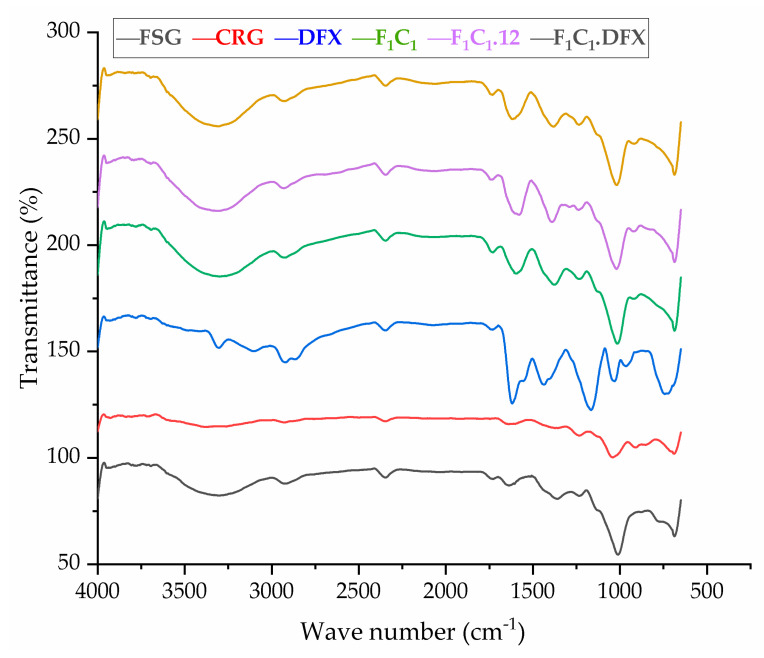
FTIR spectrum of FSG, CGN, pure DFX, F_1_C_1_, F_1_C_1_.12, and F_1_C_1_.DFX.

**Figure 5:**
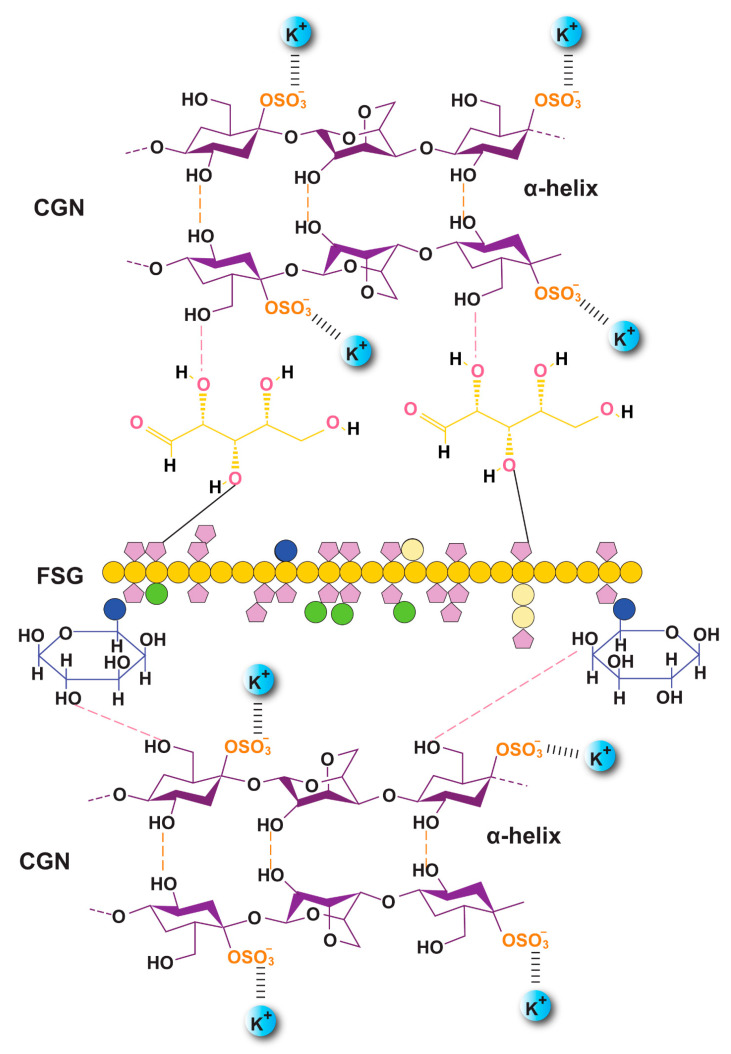
Proposed orientation of the CGN and FSG in the crosslinked biocomposite film along with probable hydrogen bonding (F_1_C_1_.12).

The FTIR spectra of the raw material and preparations are shown in Figure 4. FSG showed a major peak around 3500–3600 cm^−1^ due to -OH stretching vibration. This is due to the carboxylic acid moiety of FSG, which contributes to the high hydrophilicity of FSG. The peak at 1735 cm^−1^ was due to C=O stretching vibrations of galacturonic acid, and a peak around 1192 cm^−1^ was associated with the 3,6-anhydro-galactose bridges [13]. The pattern of CGN showed a broad band, ranging from 3100 to 3600 cm^−1^, due to a multitude of varying hydroxyl group -OH stretching, which is specific to the hydrophilicity of polysaccharides. The peaks at around 2800–3000 cm^−1^ were due to CH stretching, and those at around 795–1386– cm^−1^ (stretching) were attributed to the carbohydrate group. Peaks at 867 and 875 cm^−1^ showed the presence of the C-O-SO^3^ of D-galactose-4-sulfate, as reported by Ying and coworkers for CGN [39]. The FTIR spectrum of DFX showed a characteristic band at 1086 cm^−1^ due to N-OH stretching, specific to DFX. Moreover, signals at around 1500 and 1698 cm^−1^ were due to the stretching of N-H and C=O, respectively, hinting at the presence of amide bonds, as described in the literature [40]. The crosslinking by KCl shifted the stretching of -OH from 875–879 cm^−1^ in F_1_C_1_ to 813–883 cm^−1^ in F_1_C_1_.12. Interestingly, new peaks in F_1_C_1_.12 at 1701, 1734, and 1739 cm^−1^ were completely absent in the non-crosslinked film, and these peaks showed the shifting of C=O stretching from 1600 cm^−1^ in F_1_C_1_ due to crosslinking. Shifts in the stretching pattern from the non-crosslinked to crosslinked polysaccharides were also reported by Giz et al., 2020 [29]. Figure 5 shows the probable crosslinking and hydrogen bonding in the network of the crosslinked film. These above-mentioned peaks are clearly visible in the drug-loaded film. These results concluded that DFX was successfully encapsulated in the crosslinked blends and retained its chemical integrity.

### 2.10. Differential Scanning Calorimetry (DSC) and Thermogravimetric Analysis (TGA)

**Figure 6:**
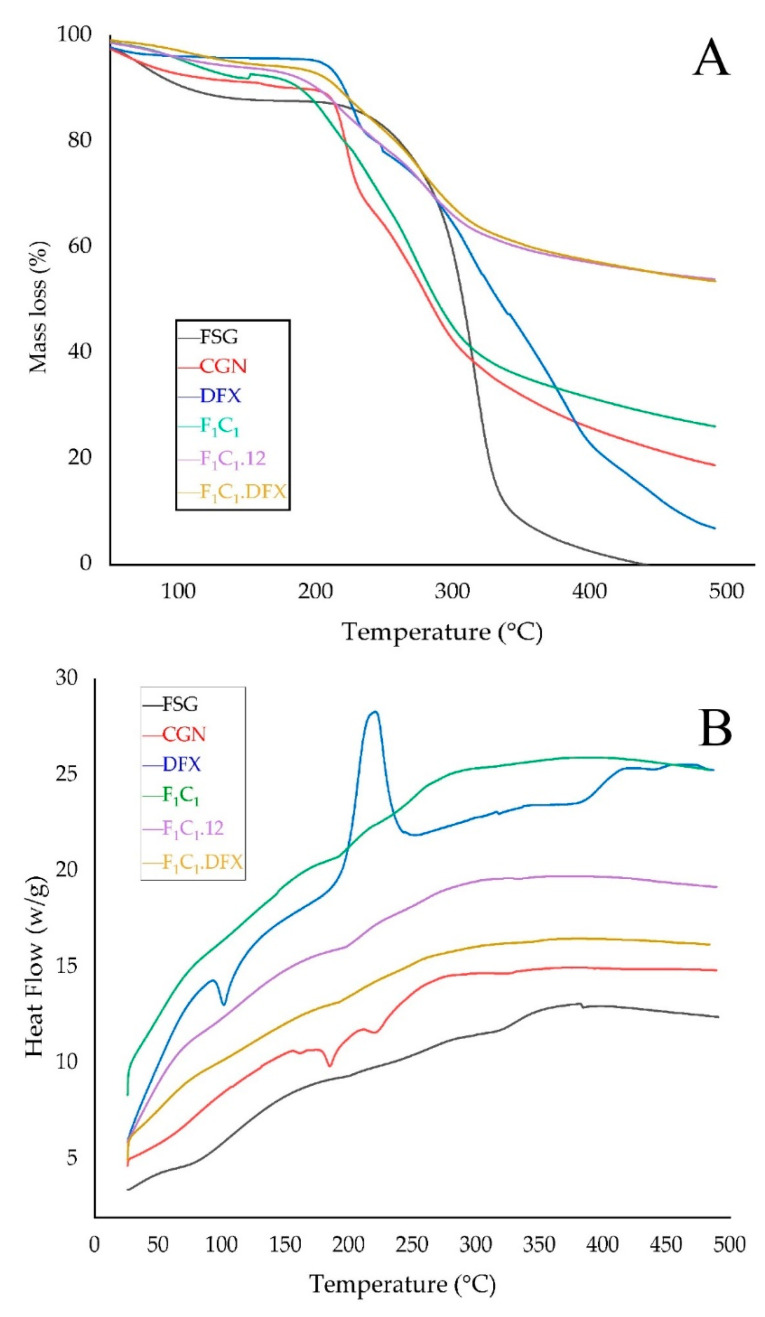
(**A**) TGA thermograms and (**B**) DSC thermograms of FSG, CGN, pure DFX, F_1_C_1_, F_1_C_1_.12, and F_1_C_1_.DFX.

TGA was used to investigate the thermal stability of the films, as shown in Figure 6A. The FSG powder exhibited a slight decrease in mass just below 100 °C; this effect is due to the evaporation of water from FSG. The maximum weight loss for FSG was observed from 200–300 °C due to the degradation of the macromolecular chains of the polysaccharides, as mentioned elsewhere [41]. A sharp peak at 280 °C and a shoulder peak at 320 °C were due to the presence of two components with different molecular weights. These two components could be an acidic and neutral fraction, as identified by Guo and coworkers [42]. In the case of the CGN powder, a loss of moisture occurred at 90 °C, whereas a sharp loss in mass was observed between 200 to 250 °C, suggesting the rapid degradation of CGN. This degradation has been attributed to the loss of the OSO^3−^ group from the polymeric backbone of CGN and carbohydrate chain fragmentation [43]. F_1_C_1_ demonstrated similar but slightly improved thermal behavior than CGN. However, a significantly improved thermal stability was observed for both F_1_C_1_.12 and F_1_C_1_.DFX due to the crosslinking of the polymeric chains by KCl. There was no effect of DFX loading on the thermal behavior of the crosslinked hydrogel film, which could be related to the molecular dispersion of the drug within the matrix of the film.

The interaction among the components of the biocomposites was assessed by changes in the melting peaks in the DSC thermograms, as shown in Figure 6B. The DSC curve of DFX showed an endothermic peak at around 100 °C, referring to the initiation of melting. DFX showed a very sharp exothermic peak at around 220 °C, indicating its crystallization; moreover, due to the sharpness and height of the peak, it can be inferred that DFX quickly recrystallized at this temperature [44]. No such peaks for DFX were evident in F_1_C_1_.DFX, implying that the thermotropic state of the composites was not altered and that the drug was encapsulated successfully in the molecularly stabilized state.

### 2.11. Study of Drug Release

**Figure 7:**
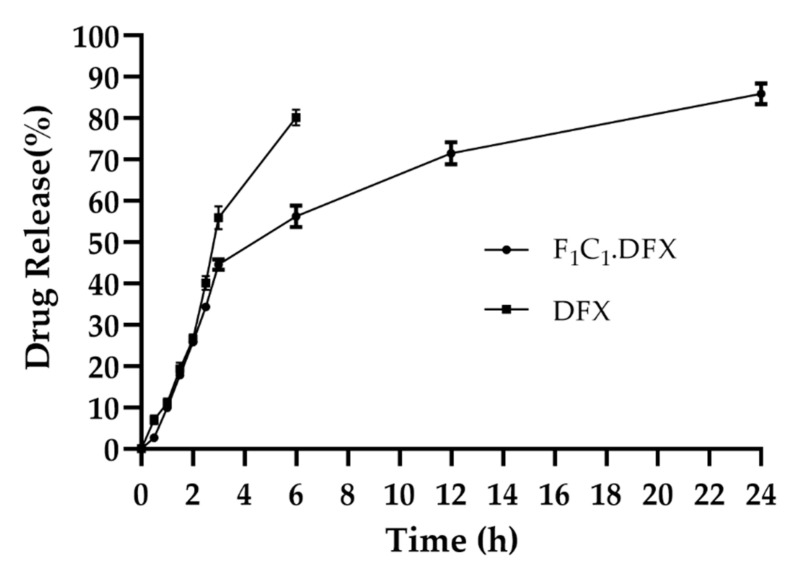
Release of DFX from F_1_C_1_.DFX in PBS (pH 7.2) at 37 °C. Data presented as mean ± SD.

One of the objectives of the design of the biocomposites was to control the release of DFX to yield the optimized effect for wound healing. The combination of FSG and CGN provides an effective semi-interpenetrating network for the sustained delivery of DFX. Both polymers were useful in controlling the release of the drugs under different formulations; however, this combination has not been reported to date. FSG has been used in oral formulations to sustain the release of moxifloxacin and fluroquinolones antibiotics [45]. Synytsya et al., 2020, used FSG for the delivery of bioactive peptides to a wound site [46]. CGN possesses suitable physicochemical properties, such as higher molecular weight and increased viscosity and gelling properties to extend the release of different active ingredients [47]. CGN has been reported to control the release of different moieties, such as vascular endothelial growth factor [48], platelet derived growth factors [49], and *Cryphaea heteromalla* (Hedw.) D. Mohr [50]. All these studies strengthen the combinational use of FSG and CGN to provide synergistic effects for the controlled delivery of DFX. It is evident from Figure 7 that the initial burst release of DFX was followed by a release in a sustained manner. The initial burst release of DFX has also been reported previously [51] and was linked to the smaller molecular weight of DFX. In a previous study, DFX hydrogels displayed an initial fast release within 1 h (about 60-70%) and then a sustained release for up to 10 h [52]. Rassu and coworkers reported that 46% of the drug was released within just 1 h and then had a delayed release of up to 48 h [53]. In another study, DFX release was controlled for up to 14 h [9]. F_1_C_1_.DFX released 40% of the drug within the first 3 h, owing to the rapid water penetration within the film, but subsequently, the drug release slowed down due to the swelling of the hydrogel matrix. Up until 24 h, DFX release reached around 80%. Hence, this biocomposite hydrogel film could provide sufficient drug coverage to the wound bed, which is necessary for healing due to a controlled drug release profile.

### 2.12. In-Vivo Wound Healing Assay

**Figure 8:**
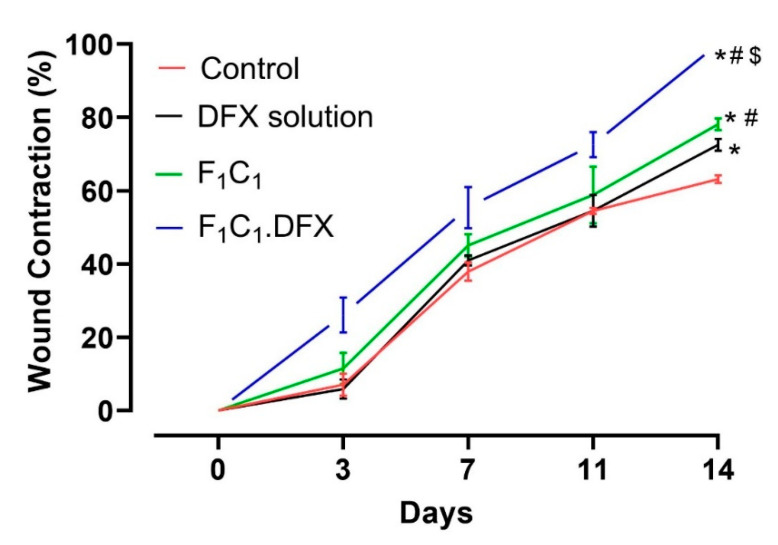
Wound contraction studies in a rat model, *n* = 4, mean ± SD. * indicates *p* < 0.05 vs. Control; # indicates *p* < 0.05 vs. DFX solution; $ indicates *p* < 0.05 vs F_1_C_1_.

In order to assess the ability of the designed biocomposite hydrogel film to expedite the wound healing process, a wound contraction analysis was carried out via a full-thickness excisional wound model. Excisional wounds are one of the most commonly used wound-healing models and are considered to resemble acute clinical wounds. These wounds are generated by the surgical removal of all skin layers (epidermis, dermis, and subcutaneous fat) from the animal. The full-thickness excisional wound model allows for the investigation of hemorrhage, inflammation, granulation tissue formation, re-epithelialization, angiogenesis, and remodeling. In this study, full-thickness wounds were created via a 6 mm biopsy punch, ensuring the complete removal of the skin layers; the healing of the full-thickness wound model in this study refers to 100% wound contraction without any scar formation [54]. To quantify the wound contraction, the wound size was determined by a digital vernier caliper, and the contraction percentage of the wounds in each group was calculated on days 0, 3, 7, 11, and 14. A more pronounced difference in the wound healing in all groups was evident after day 4 due to the proliferative phase of wound healing [55]. From Figure 8 it is evident that the wound contraction percentage was almost similar from day 0 to day 10 for animals in the untreated, DFX solution, and F_1_C_1_.12 groups. Statistically significant (*p* < 0.05) wound contractions in the animals under the drug solution and the blank film (against the untreated animals) were only observed on day 14, which showed the least wound contraction (63%). The blank film group showed better wound contraction than the drug solution group over all of the respective days of study. In addition, a significant difference in wound contraction was observed for the F_1_C_1_.12 group (78%) when compared to the DFX solution group (72%) on day 14 of the study. The better performance of F_1_C_1_.12 over the drug solution could be attributed to the hemostasis attributes of FSG [35] and the antimicrobial and anti-inflammatory properties of CGN [16]. Moreover, the hydrogel film could also have promoted wound healing by absorbing the wound exudates due to its excellent swelling properties. F_1_C_1_.DFX displayed significantly superior wound contraction (*p* < 0.05) at every observation time point, and 100% wound closure was observed for all the animals on day 14. Scar formation was evident on day 7, which disappeared, leaving no mark by day 14. Irritation to the skin (rashes, redness, and swelling within and around the wound bed) was also observed for the DFX solution, as already reported [56], but was not seen in the animals of the F_1_C_1_ and F_1_C_1_.DFX groups. This iterates that this biocomposite hydrogel film is biocompatible in nature and prevents drug-associated irritation due to controlled drug release, which also contributes to efficient wound recovery. Our results are in accordance with those previously published in the literature [8,57].

**Figure 9:**
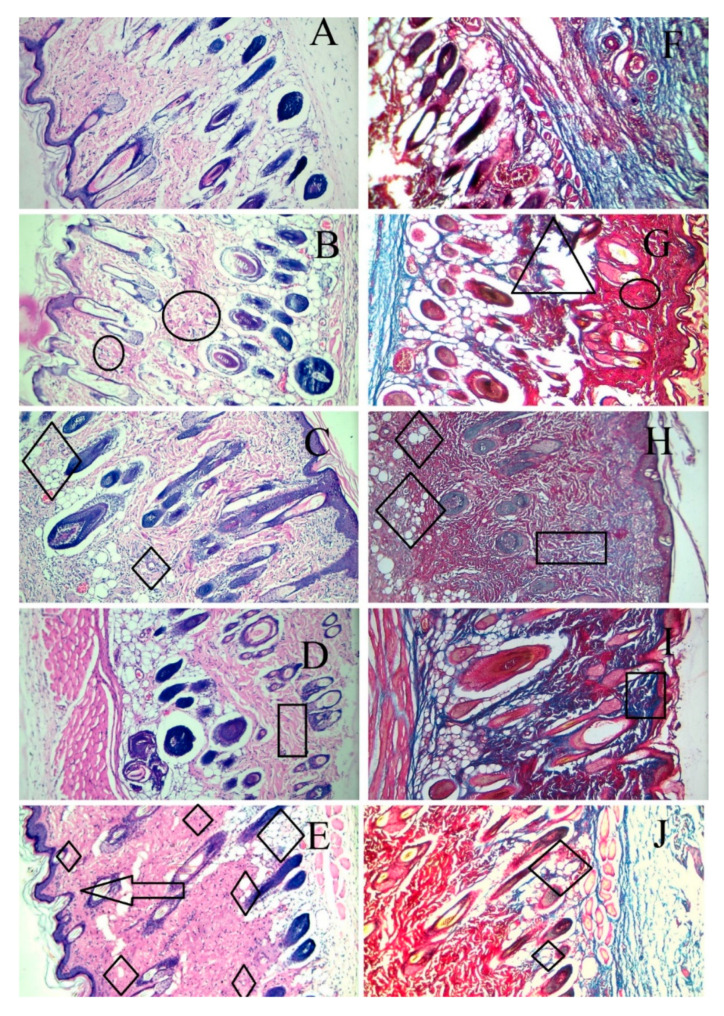
Histological evaluation by H & E and Masson trichome staining of the cutaneous wounds in the rats on the 14th day of treatment. (**A**,**F**) (healthy rats), (**B**,**G**) (wounded without intervention), (**C**,**H**) (F_1_C_1_), (**D**,**I**) (1% DFX solution), (**E**,**F**) (F_1_C_1_.DFX); (**A**–**E**) for H & E staining, (**F**–**J**) for Masson trichome staining (Δ = Ulceration, Ο = inflammation, ◊ = angiogenesis, □ = Proliferation of fibroblasts, ―> = reepithelization).

Skin tissue sections from the wound area were obtained from each animal after euthanasia and were stained by H & E and Masson trichome staining on the 14th day. As shown in Figure 9, the wounds without intervention (B and G) did not heal completely on day 14 and showed areas of proliferation (of the fibroblasts) and ulceration of the tissue. The F_1_C_1_ group (C and H) had no inflammation but an occasional migration of the fibroblasts. It is important to note that, even without the drug, F_1_C_1_ promoted wound healing; however, this effect was considerably less when compared to the drug-loaded film group. This effect could be related to FSG, as one study delineated the wound-healing properties of flaxseed mucilage [58]. The wounds were not healed completely in the DFX solution group (D and I), with the evident proliferation of the fibroblasts, weaker angiogenesis, and poor reepithelization. This shows that to exploit the effect of DFX, the release of DFX should be controlled for its prolonged stay at the wound bed. Poor angiogenesis may lead to a delay in the granulation tissue formation leaving the wound bed area prone to secondary infections and further damage. DFX is known to promote angiogenesis by the induction of the hypoxia-inducible factor-1 alpha (HIF-1α) [52]. Multiple areas of angiogenesis were observed in the F_1_C_1_.DFX group (E and H), in which new blood vessel formation could be seen clearly. On the other hand, fewer new blood vessels were observed in the wound of those animals treated with the DFX solution. This observation further confirms that the controlled and continuous exposure of the wound bed to DFX ensured rapid wound healing. In addition, F_1_C_1_.DFX facilitated the migration of endothelial cells, resulting in the promotion of neogenesis. Another important aspect for wound healing is collagen deposition, which is responsible for promoting the transformation of fibroblasts into epithelial tissues. Scarless wound healing is also dependent upon the formation of collagen 1 on the wound bed [59]. Mason Trichome staining revealed a higher degree of collagen deposition and reepithelization in F_1_C_1_.DFX (shown by deep blue area); therefore, scar formation, which was evident on day 7, disappeared, leaving no mark by day 14. Therefore, the animals treated with F_1_C_1_.DFX had fully healed skin and rapid wound closure.

Table 5 summarizes the overall performance of the biocomposite film compared with the drug solution in terms of the time required for 80% drug release (T80) to reveal the controlled drug release, wound contraction (%) at day 14, and the occurrence of skin irritation.

## 3. Conclusions

In this study, biocompatible biocomposite hydrogel films were fabricated from the natural polymers FSG and CGN for the controlled local delivery of DFX to heal full-thickness wounds. The optimized preparation not only displayed high physical and mechanical properties but also provided controlled and extended release of DFX for 24 h, which manifested as an absence in the characteristic drug irritation at the wound site and a 100% wound closure in the in vivo full-thickness excisional rat models. However, there is a need to assess the biomarkers (IL-6, TNF-α, and HIF-1α) and the immunostaining assays (particularly CD31) to understand the overall role of this drug-loaded biocomposite film in controlling inflammation, angiogenesis, and neogenesis. In conclusion, F_1_C_1_.DFX, designed in our study, has the potential to become an attractive alternative to currently available wound dressings.

## 4. Materials and Methods

### 4.1. Materials

Flax seed gum (FSG) and kappa carrageenan (CGN) were purchased from XI AN ZEBANG Biological technology Co. Ltd. (Xi’an, China) and Xiamen Sinroad Industry and Trade Co. Ltd. (Xiamen, China), respectively. Glycerol (Gly) and deferoxamine (DFX) were acquired from Merck (Darmstadt, Germany) and Sigma Aldrich (St. Louis, MO, USA), respectively. All chemicals and reagents were of analytical grade and obtained from Sigma Aldrich. Deionized ultra-filtered water was used throughout this study. All the chemicals were used as received.

### 4.2. Methods

#### 4.2.1. Preparation of Biocomposite Hydrogel Films of CGN and FSG

The FSG and CGN composite films were prepared by solvent casting method. FSG was dissolved mechanically for 10 min along with 25% glycerol until a homogenous solution was formed [13]. CGN was dissolved in water by magnetic stirrer mixing at 70 °C for 20 min. FSG solution with glycerol was added slowly into the CGN solution and further agitated for 15 min. Different blends were prepared by keeping the glycerol concentration constant; the quantities and proportion of the blends are shown in Table 1. A hot solution was poured carefully on 80 mm petri plates and dried at room temperature (25 °C) for 72 h.

Crosslinked films were prepared by employing 7% *w*/*v* solution of KCl as a crosslinker via the immersion method. After the gelation of the films, 5 mL of crosslinker solution was poured, and films of a varying crosslinking degree were prepared by choosing the time of immersion of the films in crosslinker solution for 1, 12, or 24 h. Afterwards, the films were washed repeatedly with ultrapure water and dried at room temperature. After drying, the films were evaluated visually to examine any physical defects [34]. For the drug-loaded films, DFX was dissolved in the FSG solution; the rest of the procedure followed the same as mentioned above for blank films.

#### 4.2.2. Film Thickness

Measurement of thickness was carried out by a method reported elsewhere [21]. The thickness of each film was determined using a digital micrometer (0–25 mm, 1 μm sensitivity) at 10 randomly selected points of the film. The mean value of these measurements was used.

#### 4.2.3. Mass Determination

All preparations were accurately weighed on an electronic weighing balance in triplicate [60]. The study was carried out to detect inter and intra-batch variations.

#### 4.2.4. Water Solubility (WS)

Solubility determination was carried out as previously described by Prado and coworkers [13]. Three portions of film, with a diameter of 2 cm, were dried in an oven at 105 °C for 2 h. The initial dry weight of the films (*W_i_*) were determined and immersed in beakers of 30 mL distilled water. The beakers were sealed and periodically shaken for 24 h at 25±2 °C. The insoluble portion of the films were removed and dried at 105 °C for 24 h. After drying, the final weight of insoluble matter (*W_f_*) was determined. The water solubility (*S*) of each film was determined by following equation:

$$S\;\left(\%\right)=\frac{W_{i}-W_{f}}{W_{i}}\times 100$$

#### 4.2.5. Water Vapor Transmission Rate (WVTR)

WVTR was used to determine the permeability of films towards moisture by using a previously reported method [61]. Each film was cut into a circular shape and mounted on a cylindrical tube, with a diameter of 2 cm, containing 30 mL of deionized water. After initial weighing (*W_i_*), the whole apparatus was incubated at 37 °C for 24 h. After 24 h, the mass of the bottle was noted (*W_f_*). WVTR was calculated by a similar method, as reported in earlier studies [28,62,63].

WVTR (gm2day)=Wi−WfArea of the film ×24

#### 4.2.6. Swelling Study

Degree of swelling was measured gravimetrically [34] by monitoring weight changes due to water uptake as a function of time. Hydrogel films were cut into 1×1 cm dimensions and weighed accurately by using an analytical balance. After weighing, the films were placed carefully in 5 mL of deionized water. The samples were taken out at different time intervals of 15 min, 30 min, 1 h, 3 h, 6 h, 12 h, and 24 h. The water was replaced after each measurement. The percentage of swelling rate was calculated by following equation:

$$SR\;\left(\%\right)=\frac{W_{i}-W_{0}}{W_{0}}\times 100$$

where *W_i_* is the mass of the swollen sample and *W_0_* is the initial weight of dry film.

#### 4.2.7. Determination of Encapsulation Efficiency (EE%)

EE (%) was determined by employing a previously reported method with minor modifications [44]. The DFX-loaded films were weighed, and a homogenous solution was added into centrifugal-ultrafiltration tubes (Microcon MWCO 3000, Millipore Co, Burlington, MA, USA) and centrifuged at 15,000 rpm for 30 min. The amount of free DFO in the supernatant was measured by UV-Vis spectrophotometer at 230 nm from an already constructed calibration curve. EE (%) was then determined by using following equation:

$$EE\;\left(\%\right)=\frac{Weight\;of\;loaded\;DFX}{Weight\;of\;feed\;DFX}\times 100$$

#### 4.2.8. Mechanical Properties of Composites

The folding endurance (FE) of the films was measured according to a method reported by Shahzad and coworkers [27]. The films were cut into equal areas and folded on the same point, until the films broke. The number of folds before breakage was recorded as the FE. The tensile strength (TS) at break, elongation at break (EAB), and Young’s modulus (YM) were determined from stress-strain data obtained through a dynamic mechanical analyzer (Q800, TA Instruments, New Castle, DE, USA). Each test was repeated in triplicate. Experiments were performed at 24 °C and a relative humidity (RH) level of 38 ± 1%. Temperature and RH levels were monitored by taking measurements every 15 min throughout each experiment [41].

#### 4.2.9. Scanning Electron Microscopy (SEM)

The morphology of the films was pictured using a scanning electron microscope (model TM3030, Hitachi, Tokyo, Japan), operating at 5 kV. The samples were mounted on an aluminum stub using a double-sided tape and then coated with a gold layer [21].

#### 4.2.10. Fourier Transform Infrared Spectroscopy (FTIR)

FTIR spectra were recorded by ATR-FTIR (Jasco model FT/IR-410, 420 Herschel series—Jasco Corporation, Tokyo, Japan). The experiments were carried out in the range of 4000–500 cm^−1^ with a spectral resolution of 2 cm^−1^ [13,31,34].

#### 4.2.11. Thermogravimetric Analysis (TGA) and Differential Scanning Calorimetry (DSC)

TGA of the samples was assessed using a Shimadzu DTG-60H equipment (Kyoto, Japan). The analysis was performed on the samples weighing 6 mg in a nitrogen atmosphere with a flow rate of 50 mL/min, at a heating rate of 10 °C/min and a temperature range of 25–500 °C [13]. The thermal behavior of the raw material and films was analyzed by differential scanning calorimeter (Shimadzu DSC-60, Kyoto, Japan). Samples of 3 mg were heated in the aluminum pan at a temperature range of 25–500 °C under a nitrogen flow rate of 20 mL/min and heated at a scanning rate of 20 °C/min [27].

#### 4.2.12. Study of Drug Release

The in vitro drug release study of DFX-loaded films was carried out by following a previously established method with slight modifications. The drug release study was performed via the dialysis method. In this method, a membrane-bound tube containing 5 mL of PBS was partially immersed in a beaker containing 50 mL of PBS. The drug or film samples were immersed in the membrane-bound tube; the whole apparatus was placed on magnetic stirrer at 300 rpm. At predetermined time intervals, 10 mL of PBS was withdrawn from beaker and analyzed spectrophotometrically. The medium in beaker was replaced by freshly prepared 10 mL of PBS. The procedure was performed in triplicate [64].

#### 4.2.13. In-Vivo Wound Healing Assay

All animal experiments were approved by the Ethical Review committee of Government College University Faisalabad under the reference number of GCUF/ERC/59. For the study, 20 adult male rats, weighing 170–190 g, were obtained from the animal facility of the Faculty of Pharmaceutical Sciences, Government College University Faisalabad (Faisalabad, Pakistan). The rats were given free access to water and food with 12 h light and 12 h dark cycles under temperature conditions of 24 –25 °C throughout the study. The rats were kept in the animal facility for 2 weeks prior to study so that they could be acclimatized properly before study. The rats were then randomly distributed into five groups, and each group was comprised of 4 animals (*n* = 4) and specified as (a) Healthy Group, (b) Diseased Group or control, (c) 1% DFX solution, (d) blank film (F_1_C_1_.12), and (e) drug-loaded film (F_1_C_1_.DFX).

The rats were anesthetized by 50 mg/kg of ketamine and 5 mg/kg xylazine [55]. The dorsal surface of the rats were depilated, and excisional wounds on the dorsum of the rats were created by 6 mm biopsy punch [65]. The healthy group was left without wounds, with the animals of all the other groups being inflicted with wounds. The diseased group or control animals were left untreated. The other 3 groups were treated as mentioned above. Before the application of the films, the wounds were washed with normal saline and then treated accordingly. Wounds were bandaged by sterile gauze to prevent the removal of the dressing by scratching and biting by the rats.

The films were changed daily, and the progress of wound healing was assessed by measuring the wound size by a digital vernier caliper on days 0, 1, 3, 7 10 and 14. Percentage of wound contraction was determined by the formula mentioned in earlier studies [9,55,66,67].

$$Wound\;contraction\;\left(\%\right)=\frac{A^{o}-A_{i}}{A^{o}}\times 100$$

where *A*° is initial area of the wound at day 0, and *A_i_* is the area of wound at a specific day.

Animals were euthanized on day 14, and the histological analyses were performed by removing the wound site and the adjacent normal skin, which were then soaked in 10% paraformaldehyde. The samples were fixed in paraffin to prepare slices of 5 µm. The sections were stained with hematoxylin-eosin (H & E) and Masson trichrome to evaluate the different stages of the wound, such as angiogenesis, wound reepithelization, and collagen deposition.

### 4.3. Statistical Analysis

Values are presented as means ± SD. One-way ANOVA was employed with post-hoc Tukey test for multiple comparison. *p* values < 0.05 were considered statistically significant.

## Figures and Tables

**Table 1:** Composition of the non-crosslinked and crosslinked biocomposite hydrogel films.

Samples	FSG % (*w*/*v*)	CGN % (*w*/*v*)	Immersion Time (h)
**F_1_C_1_**	1	1	0
**F_1_C_1._1**	1	1	1
**F_1_C_1._12**	1	1	12
**F_1_C_1._24**	1	1	24
**F_1.5_C_0.5_**	1.5	0.5	0
**F_1.5_C_0.5._1**	1.5	0.5	1
**F_1.5_C_0.5._12**	1.5	0.5	12
**F_1.5_C_0.5._24**	1.5	0.5	24
**C_1.5_F_0.5_**	0.5	1.5	0
**C_1.5_F_0.5._1**	0.5	1.5	1
**C_1.5_F_0.5._12**	0.5	1.5	12
**C_1.5_F_0.5._24**	0.5	1.5	24

**Table 2:** Water solubility (WS), Water vapor transmission rate (WVTR), and Flatness study.

Composites	Thickness (mm)	Weight-Variation (g)	WS (%)	WVTR (g/m^2^·day)	Flatness %
**F_1_C_1_**	0.05 ± 0.005	0.02 ± 0.002	100 ± 0.00	859.87 ± 31.85	100
**F_1_C_1_.1**	0.11 ± 0.006	0.04 ± 0.004	79.85 ± 1.36	764.33 ± 31.85	100
**F_1_C_1_.12**	0.13 ± 0.005	0.06 ± 0.088	60.67 ± 1.15	732.48 ± 31.85	100
**F_1_C_1_.24**	0.17 ± 0.005	0.06 ± 0.002	65.56 ± 1.93	1591.51 ± 63.69	100
**F_1.5_.C_0.5_**	0.05 ± 0.005	0.03 ± 0.004	100.00 ± 0.00	955.41 ± 31.85	100
**F_1.5_.C_0.5_.1**	0.07 ± 0.004	0.03 ± 0.005	75.00 ± 0.00	1146.50 ± 63.69	100
**F_1.5_.C_0.5_.12**	0.07 ± 0.010	0.03 ± 0.003	74.17 ± 1.44	1433.12 ± 31.85	100
**F_1.5_.C_0.5_.24**	0.12 ± 0.004	0.05 ± 0.006	59.33 ± 1.15	1337.58 ± 31.85	100
**C_1.5_F_0.5_**	0.05 ± 0.004	0.03 ± 0.002	100 ± 0.00	849.26 ± 18.39	100
**C_1.5_F_0.5_.1**	0.17 ± 0.004	0.03 ± 0.003	49.17 ± 1.44	774.95 ± 18.39	100
**C_1.5_F_0.5_.12**	0.17 ± 0.004	0.05 ± 0.003	59.33 ± 1.15	721.87 ± 18.39	100
**C_1.5_F_0.5_.24**	0.18 ± 0.004	0.05 ± 0.003	60.00 ± 0.00	881.10 ± 18.39	100

**Table 3:** EE (%) of F_1_C_1_.12 films at varying concentrations of DFX.

Sample	EE (%)
**0.5% DFX**	26.86 ± 0.91
**1% DFX**	83.39 ± 1.29
**2% DFX**	40.99 ± 0.59

**Table 4:** Mechanical properties of the biocomposite hydrogel films.

Hydrogel Films	FE	TS (MPa)	EAB (%)	YM (MPa/mm^2^)
**F_1_C_1_**	21.00 ± 0.816	7.52 ± 0.07	43.71 ± 0.029	2.72 ± 0.06
**F_1_C_1_.12**	19.66 ± 0.471	19.51 ± 0.032	28.41 ± 0.191	0.48 ± 0.053
**F_1_C_1_.DFX**	21.00 ± 0.800	20.20 ± 0.013	116.69 ± 0.08	0.17 ± 0.017

**Table 5:** Comparative performance of DFX solution and F_1_C_1_.DFX.

Formulation	T80 (Hours)	Wound Contraction at Day 14 (%)	Skin Irritation
DFX solution	6	72%	Yes
F_1_C_1_.DFX	24	100%	No

## Data Availability

Most of the data is presented in the manuscript. Raw data cannot be shared at the moment due to the technical and privacy constraints, but it is available on demand from the corresponding author.
